# MLVA polymorphism of *Salmonella enterica *subspecies isolated from humans, animals, and food in Cambodia

**DOI:** 10.1186/1756-0500-4-306

**Published:** 2011-08-24

**Authors:** Hélène van Cuyck, Alexandra Farbos-Granger, Philippe Leroy, Vuthy Yith, Bertrand Guillard, Jean Louis Sarthou, Jean Louis Koeck, Sun Lay Kruy

**Affiliations:** 1 Hôpital d'Instruction des Armées Robert Picqué, Service de Biologie Clinique, Villenave d'Ornon Bordeaux, France; 2 Institut Pasteur du Cambodge, 5 Bd Monivong, BP 983, Phnom Penh, Cambodia

## Abstract

**Background:**

*Salmonella *(*S*.) *enterica *is the main cause of salmonellosis in humans and animals. The epidemiology of this infection involves large geographical distances, and strains related to an episode of salmonellosis therefore need to be reliably discriminated. Due to the limitations of serotyping, molecular genotyping methods have been developed, including multiple loci variable number of tandem repeats (VNTR) analysis (MLVA). In our study, 11 variable number tandem-repeats markers were selected from the *S. enterica *Typhimurium LT2 genome to evaluate the genetic diversity of 206 *S. enterica *strains collected in Cambodia between 2001 and 2007.

**Findings:**

Thirty one serovars were identified from three sources: humans, animals and food. The markers were able to discriminate all strains from 2 to 17 alleles. Using the genotype phylogeny repartition, MLVA distinguished 107 genotypes clustered into two main groups: *S. enterica *Typhi and other serovars. Four serovars (Derby, Schwarzengrund, Stanley, and Weltevreden) were dispersed in 2 to 5 phylogenic branches. Allelic variations within *S. enterica *serovars was represented using the minimum spanning tree. For several genotypes, we identified clonal complexes within the serovars. This finding supports the notion of endemo-epidemic diffusion within animals, food, or humans. Furthermore, a clonal transmission from one source to another was reported. Four markers (STTR3, STTR5, STTR8, and Sal20) presented a high diversity index (DI > 0.80).

**Conclusions:**

In summary, MLVA can be used in the typing and genetic profiling of a large diversity of *S. enterica *serovars, as well as determining the epidemiological relationships of the strains with the geography of the area.

## Findings

Complete genome sequences are now available for many bacteria, including some *S. enterica *strains. These can be used to determine DNA repeat-motif sequences through multiple-loci variable-number of tandem repeats (VNTR) analysis (MLVA), which has a high capacity for discrimination [1-5]. A database of tandem repeats for several completed *Salmonella *genome sequences is available http://minisatellites.u-psud.fr[6].

Using *S. enterica *Typhi, *S. enterica *Typhimurium, *S. enterica *Enteritidis, and *S. enterica *Newport as reference strains, a large panel of VNTRs markers using MLVA technique tested different serovars of *S. enterica *using human and environmental samples from different geographic origins [1,3,4,7-9]. To date, 58 VNTRs markers have been described, but only a few of these are still being used [2]. These have been developed from the genotyping and phylogenetic analyses of the *S. enterica *serovars Typhimurium [3,4], Typhi [8,10], and Enteritidis [11,12] and Newport [9].

In the study, we investigated the genetic distribution of 31 *Salmonella enterica *serovars from different sources (human, animal, and food) using MLVA. This was done by selecting a set of 11 VNTR loci [1,2,8] that were tested to determine the genetic diversity of strains isolated in Phnom Penh, Cambodia. The choice of the VNTR loci and the multiplicity of the serovars may contribute to the evaluation of the genetic framework of the *Salmonella *genus. Furthermore, sequence analysis of VNTR loci was employed to investigate different patterns of tandem repeats.

The set of 11 VNTR loci was used to discriminate 31 serovars represented by 206 isolates. The LT2 reference DNA, tested as an internal control, gave identical expected VNTR fragments with the repeat motifs, thus demonstrating the reproducibility of the system.

With the Sal06 marker, PCR amplification was negative for 3 out of 5 Albany isolates, 3 out of 7 Braenderup isolates, 4 out of 6 Choleraesuis isolates, 3 out of 9 Corvallis isolates, 6 out of 8 Hvittingfos isolates, 2 out of 2 Kentucky isolates, and 1 out of 4 Ohio isolates. PCR for the Sal23 and STTR8 markers were negative for the Choleraesuis serovar and PCR for the STTR8 marker was negative for the Mbandaka serovar. All Typhi isolates were differentiated from the other serovars by the Sal06 and TR1 markers. The Sal23 marker distinguished the Albany, Braenderup, Corvallis, and Typhi serovars from the others.

### Composition of the amplicons

To validate the organization of amplified fragments having an unexpected TR length, DNA sequence analysis was performed for the following markers: Sal06, Sal15, Sal20, STTR3, STTR5, STTR7, and STTR8. Fifty fragments were amplified from samples corresponding to 16 serovars and the sequences obtained were compared to the fragments of LT2 reference strain. A TR sequence greater than or equal to 0.5 repeat copies was considered as one copy and a repeat copy less than 0.5 was considered as a zero-repeat copy. All loci without amplification or presenting amplifications without TR still need to be noted and are considered as "0" alleles in the analysis [5,13].

**Figure 1:**
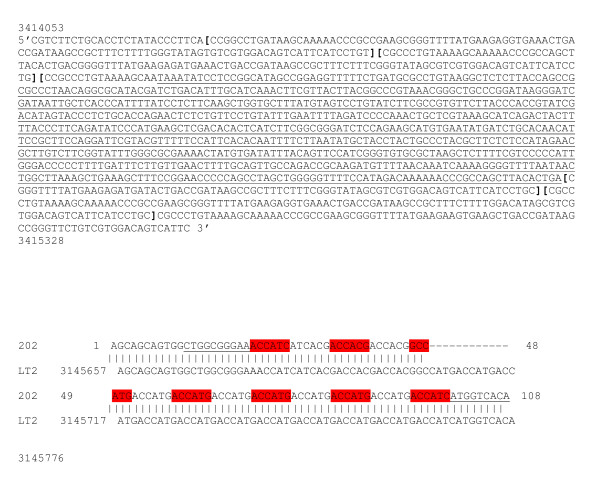
**Validation of marker and a new allele by sequencing of the PCR fragment**. **Figure 1A: **S. enterica Sandiego (SA_01_06A) sequence obtained using STTR8 marker. The sequences between brackets are TR sequences. The inserted sequence is underlined. Position of the fragment was obtained by comparison of the sequence with the S.enterica Typhimurium LT2 reference strain using BLAST program in ncbi. **Figure 1B**: A new allele; *S.enterica *Paratyphi B consensus sequence of the n° 202 (SA_03_01B) isolate obtained using the STTR5 marker. The TR motif is ACCACG. The underlined sequences are flanking sequences of the repeat motif, colored in red and grey alternatively. The sequence is composed of 13 TR. Flanking sequences are underlying. The comparison of sequences between *S.enterica *Paratyphi B n° 202 (SA_03_01B) isolate and *S. enterica *Typhimurium LT2 (259_15U_6bp) reference strain.

There were three possible compositions of VNTR loci: (i) DNA fragments harboring expected repeat motifs, (ii) DNA fragments of unexpected length with insertion or deletion within the motif, or (iii) DNA fragments of unexpected length with repeat motifs (Figure 1). The *S. enterica *Sandiego (SA_01_06A) presented four units in the STTR8 VNTR with an insertion of a 733 bp length fragment in the third one (Figure 1A). In addition, one Paratyphi B n° 202 (SA_03_01B) isolate presented with 13 TR in the STTR5 VNTR (Figure 1B), whereas the *Salmonella *Paratyphi B SBP7 reference strain in http://minisatellites.u-psud.fr presented only two matches left at 3,145,646 and 3,022,019 genome positions but no matches right with the STTR5 VNTR.

### Diversity index

Allelic variability for all strains and sources was assessed for 11 markers using MLVA. VNTR loci displayed a wide range of polymorphism ranging from 2 to 17 alleles (Table 1). The global index of diversity was closed to one (DI = 0.988), indicating that the 11 markers had a high discriminatory effect. A high global diversity index was found for all isolates from three sources indicating a high discriminatory effect between sources: food DI = 0.985, animal DI = 0.985 and human DI = 0.894.

**Table 1:** Genetic diversity of 206 *S.enterica *isolates assessed by MLVA using 11 markers

Locus (alias)	Reference strain _genome position_ repeat (bp) _ copy number	Number of alleles	Allele size range in bp	HGDI^a^			
				
				All (n = 206)	Human (n = 45)	Animal (n = 133)	Food (n = 28)
Sal06	LT2_789139_6bp_3U	3	3, 5 (162 - 174)	0.463	0.524	0.200	0.359
Sal10	LT2_2053093_12bp_3U	3	1, 2, 3 (184 - 208)	0.128	0.044	0.178	0.000
Sal15	LT2_3067414_12bp_3U	3	2, 3 (189 - 201)	0.367	0.411	0.139	0.313
Sal20	LT2_4301685_3bp_10U	11	10,12, 13, 15, 16, 17, 18, 19, 20, 21, 24 (175 - 217)	0.805	0.627	0.743	0.761
Sal23	LT2_4774034_12bp_3U	3	3, 4 (250 - 262)	0.520	0.244	0.485	0.519
STTR3	LT2_3629458_33bp_14U	12	9, 10, 11.5, 12, 13, 14, 15, 16, 17, 18, 22 (325 - 754)	0.828	0.548	0.782	0.815
STTR5 (Sal16)	LT2_3184503_6bp_15U	17	6, 8, 9, 10, 11, 12, 13, 14, 15, 16, 17, 19, 20, 21, 22, 23, 25 (205 - 319)	0.882	0.855	0.845	0.915
STTR7	LT2_1039431_39bp_8U	8	2, 5, 6, 7, 8, 9, 10 (360 - 672)	0.739	0.455	0.779	0.735
STTR8	LT2_3414008_116bp_7U	10	2, 3, 4, 5, 6, 7,10 (345 - 1273)	0.830	0.551	0.830	0.755
TR1 (Sal 11)	LT2_2053579_7bp_3U	2	3, 5 (191 - 205)	0.243	0.469	0.000	0.000
TR5(Sal 22)	LT2_4645085_7bp_3U	2	3, 4 (173 - 180)	0.243	0.469	0.000	0.000
Total				0.988	0.894	0.985	0.985
Genotypes				107	16	75	24

^a ^Hunter and Gaston Diversity Index

Four out of the 11 markers (Sal20, STTR3, STTR5, and STTR8)that were tested in this study were highly polymorphic for the 31 *Salmonella *serovars and discriminatory for all strains (DI > 0.80). A large allelic-diversity range was observed for all strains using Sal20, STTR3, STTR5, and STTR8. The STTR5 in *yohM *gene had short repeat copies (6 bp) and showed the highest allelic size variation with 17 alleles ranging from 6 to 25 repeat motifs. This marker had also a high discriminatory power for all of the isolates (DI = 0.882) regardless of their origin; food DI = 0.915, human DI = 0.855, and animal DI = 0.845. The STTR3, STTR8, and Sal 20 markers were poorly discriminant for human isolates (DI < 0.8). The STTR3 marker was more discriminant for the food isolates (DI = 0.815) and the STTR8 marker was more discriminant for the animal isolates (DI = 0.830). The Sal20 marker was more discriminant for the whole population studied (DI = 0.805) than for each origin (0.627 for human isolates, 0.743 for animal isolates, and 0.761 for food isolate). Eight other markers Sal06, Sal10, Sal15, Sal23, STTR7, TR1 (Sal11), and TR5 (Sal22) were less discriminant, whatever the origin of isolates.

The isolates were distributed into 75 genotypes for 133 animal isolates, 24 genotypes for 28 food isolates, and 16 genotypes for 45 human isolates. The isolate population from food was the most diverse, with almost one genotype per isolate, followed by the isolate population from animals with one genotype for two isolates, and then the human isolate population with one genotype for four isolates.

### Dendrogram and minimum spanning tree

The categorical coefficient and unweighted pair group method with arithmetic average (UPGMA) were used to generate a dendrogram.

**Figure 2:**
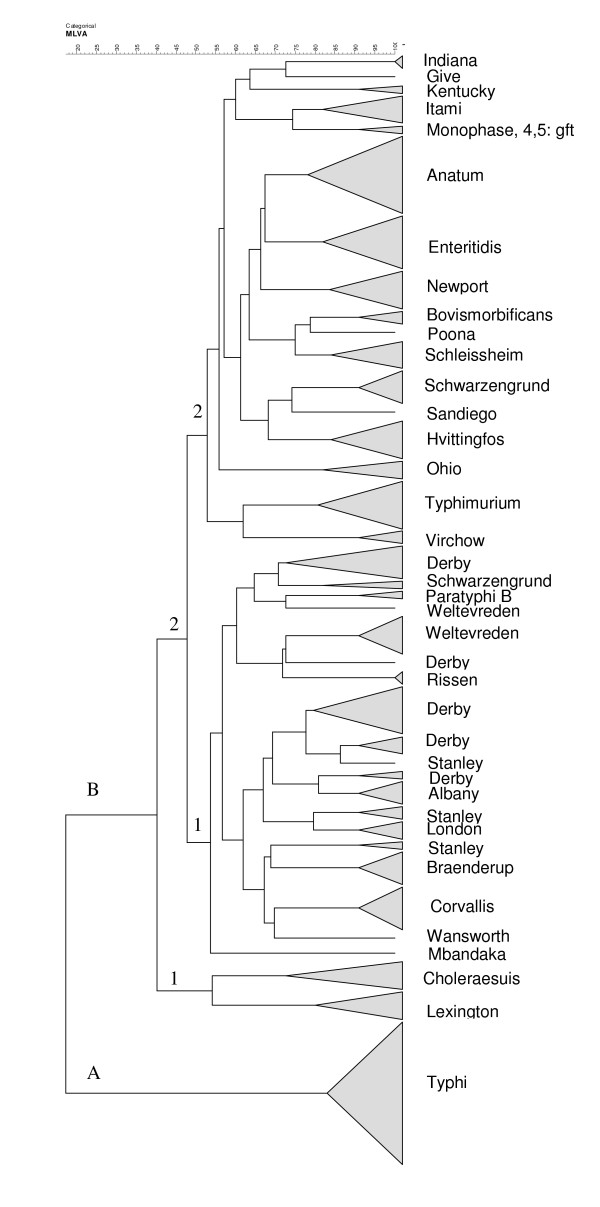
**MLVA types distribution of 31 *Salmonella enterica *serovars using the categorical coefficient and the cluster analysis pairwise similarities (UPGMA)**. The 206 isolates studied were obtained from three sources: humans (medical microbiology laboratory, 2004-2007, IPC and National Pediatric Hospital, 2005), animals (a study of food microbiology laboratory, IPC and Animal Health Department, Ministry of Agriculture, Forest and Fishery, Cambodia, 2003-2004) and from the Action de Concertation Inter-Pasteurienne (poulet project, 2006-2007) and food (food microbiology laboratory, 2001-2007, IPC).

The genetic clone *S. enterica *serovar Typhi which was exclusively from humans and rather homogeneous, diverged from the main group B clustering of 30 *Salmonella enterica *serovars. group B contains two clonal subgroups, B1 and B2, which are independent from the origin of the isolates. The B1 subgroup included the Choleraesuis and Lexington serovars. The B2 subgroup was genetically more complex with 28 serovars clustered into subclonal groups B2.1 which included 11 serovars and B2.2 which included 17 serovars (Figure 2).

The Schwarzengrund serovar is dispersed in both the B2.1 and B2.2 clusters. With the exception of the Mbandaka unique strain, two distinct unequal populations of genotypes can be distinguished in the B2.1 subgroup. Within this subgroup, the *S. enterica *Weltevreden, Derby, and Stanley serovars present several subclones. The B2.2 subgroup is more heterogeneous. These results show that the antigen profile occurs in isolates belonging to several distinct clonal lineages with different evolutionary patterns.

**Figure 3:**
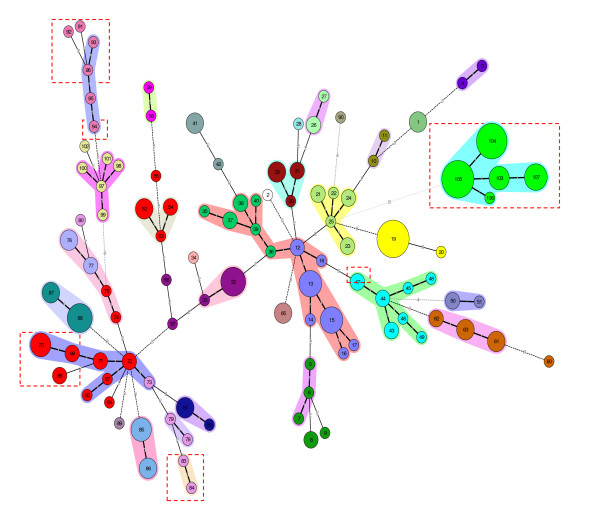
**Minimum spanning trees obtained using 11 markers for the polymorphism profile of 206 *Salmonella enterica *subspecies strains discriminated into 107 MLVA types**. The MST construction was made using the categorical coefficient. Each circle represents a different MLVA type. Each serovar is shaded with a different color. The size of the circle reflects the number of isolates whereas the distance between the circles represents the genetic divergence (heavy short lines connect SLVs, thin long lines connect DLVs, and dotted lines indicate the connection between 2 allelic distribution types). The number in each circle indicates the genotype. Red dotted squares indicate human isolates. The halos surrounding the various types denote the groupings obtained by Bionumerics analysis. In Bionumerics MLVA clonal complexes are grouping genotypes different by one allele.

On the basis of the composition of allelic distribution, a phylogenic modeling of the strains using MLVA was deduced by the construction of a minimum spanning tree (MST) (Figure 3). The 206 isolates (31 serovars) were distributed into 107 genotypes, although MLVA is more discriminant than serotyping. There is a good correlation between genotypes and serotypes, and two identical genotypes always belong to the same serotype. The degree of diversification was different depending on the serovar. The genotypes differing by one single locus variation (SLV) were clustered. The one-marker difference created clonal complexes which grouped one or several serovars. For example, Corvallis, Bovismordificans, Braenderup, Kentucky, London, Monophasic 4, 5: fgt, Typhi, Paratyphi B, Schleissheim, Typhimurium, and Virchow serovars, were not very diverged and constituted a serovar clonal complex with one SLV. The human *Salmonella *Typhi clonal complex differed by eight loci from the *Salmonella *Newport serovar (genotype n^o^25). Clones complexes from the same serovar, such as the Stanley serovar, can be grouped in two separated clonal complexes according to their origin (human or food). Some serovars, in particular the Derby and Stanley serovars are more diverse than others. The human isolates belonged to only five of the 31 serovars present in this study. For each of these serovars, the human isolates belonged to the same clonal complex, which also included animal and food isolates.

Five serovars (Give, Mbandaka, Poona, Sandiego, and Wansworth) are single isolates. The genetic divergence between *Salmonella enterica *serovars included in the analysis was very variable and the serovars differed by two to eight VNTR markers. Within the serovars the MLVA type between genotypes varies by one or two loci and rarely by more than three. However, subclones of *S. enterica *Derby, Schwarzengrund, Stanley, and Weltevreden showed differences in one to seven loci. These results are concordant with the pattern of the dendrogram.

MLVA is a reliable method for the molecular discrimination of *Salmonella enterica *isolates causing salmonellosis in humans and animals. The panel of VNTR loci used in this study was determined after reviewing the literature and applied to a collection of isolates obtained from different sources (animal, food or human) without any obvious connection to an outbreak.

This paper reports the MLVA results on a collection of 206 *Salmonella enterica *isolates from Phnom Penh, Cambodia. The study focused on six serovars (Anatum, Enteritidis, Newport, Paratyphi B, Typhi, and Typhimurium) previously analyzed by others using MLVA [1,3,8] and 25 untested serovars (Albany, Braenderup, Bovismorbificans, Choleraesuis, Corvallis, Derby, Give, Hvittingfos, Indiana, Itami, Kentucky, Lexington, London, Mbandaka, Ohio, Poona, Rissen, Sandiego, Scheissheim, Schwarzengrund, Stanley, Virchow, Weltevreden, Wansworth, and Monophasic 4, 5: fgt), contributing to the follow-up of the genetic diversity of *Salmonella enterica *isolates. With the exception of the Typhi serovars [8], none of the serovars that we studied have been previously analyzed by MLVA in Asia.

The sequencing of PCR bands for analysis of repeat motifs helped to validate the presence and the number of motifs in the amplified fragment and the deletions or insertions in a motif. This was particularly the case for fragments of unexpected length. In our study, the number of repetitions was concordant with allelic variations for small units (< 40 bp). An insertion within a motif for a large amplified fragment (> 900 bp) using STTR8 loci was found (Figure 1). On contrast, other studies had shown insertions at the beginning or the end of the repetition for all of the markers we tested [7]. Using the STTR8 locus, the dispersion of TR for the Typhimurium serovar discriminated two Typhimurium isolates harboring five (4.9) repeats, and eight other isolates harboring seven (6.5) repeats. These results correspond to the findings of previous studies in which four repetitions [3] or six repeats motifs [7] were reported.

STTR5, STTR3, STTR8, and Sal20 VNTRs presented with 17, 12, 10, and 11 alleles respectively. These markers showed a high degree of discrimination among all strains with a large allelic distribution. Two markers (Sal06, and Sal10) were less discriminatory than previously reported [1,3]. The TR1 marker had a lack of discrimination compared with a previous study of *S. enterica *Typhi [1]. The TR5 marker and STTR7 marker were more discriminatory than previously described [3,8]. Identical allelic results were observed for Sal15, TR5, and Sal23 loci, which was consistent with other studies [1]. The scope of the number and the diversity of the serovars studied confirmed the allelic scale of the 11 VNTR loci and allowed identification of other alleles. It is remarkable that the most discriminatory markers are involved either in the pathogen-host interaction (antigen virulence) or in the pathogen-environment (cell division) interaction.

The dendrogram findings supported a heterogeneous clone dispersion for a serovar distributed as different branches in the same group (five branches for *S. enterica *Derb*y*, three branches for *S. enterica *Stanley, and two branches for *S. enterica *Weltevreden serovars). For *S. enterica *Schwarzengrund, the distribution was in two different subgroups. Population genetic analysis of allelic variation in chromosomal structural genes has demonstrated that isolates of *Salmonella *of the same serovar are genotypically heterogeneous. For some serovars, groups of genetically distant individuals are observed, appearing as branches (dendrogram) or clonal complexes (MST). In some cases the same serovar may represent two or more highly divergent phylogenetic lineages as previously reported for the *S. enterica *Derby and *S. enterica *Newport serovars [14]. However, this may be also due to either the number of other serovars studied or the collection of isolates from a restricted area, Phnom Penh in Cambodia. In other studies the population of isolates was selected from different countries worldwide, and was therefore more dispersed [1,8]. The difference of dispersion for the *S. enterica *Typhi serovar indicated that the choice of VNTR depended on the population that was studied. In a homogeneous population including few other serovars [1,3,8] a discriminator VNTR should be chosen, whereas in a much diversified serovar population, a panel of discriminant and non- discriminant VNTRs has to be selected.

The representation of the MLVA results by using MST dispersed heterogeneous genetic variations of *Salmonella *isolates within the same serovar in SLVs and DLVs as previously reported [1]. Isolates obtained from different sources (human, animal, or food), are genetically linked and showed that *Salmonella *is a highly clonal organism [15]. MST constitutes a convenient tool to study the modalities of salmonella transmission between animals and humans. It would be interesting to test the hypothesis of an eventual enhanced transmissibility from animals to humans of particular genotypes belonging to the same serovar. For the same serovar, different genotypes may be associated either in a clonal complex, such as the *S. enterica *Corvallis serovars, or as a more diversified complex such as the *S. enterica *Typhimurium serovar. The genotypic dispersion of animal and food *Salmonella *populations was larger than that for human isolates. However, the minimal presence of Enteritidis and Typhimurium serovar in human isolates may reflect a bias in the population that was selected with over-representation of animal strains.

In conclusion, the MLVA assay using agarose electrophoresis is a simple, rapid, and powerful technique to genotype *S. enterica *isolates from human, food, or animal sources. The 11 markers used in this study permitted the subtyping of 107 genotypes from 206 isolates of 31 *Salmonella enterica *serovars. However, four out of the eleven studied markers presented an effective discriminatory power and should at least be included in further studies where the choice of other markers will depend on the aim of the study and the serovar being analyzed. The food population presented the largest variation in genotypes, followed by animal, and then human isolates. MLVA is an important tool for surveillance and investigation of outbreaks of human or animal salmonellosis infections.

## Competing interests

The authors declare that they have no competing interests.

## Authors' contributions

HVK: participated in the sequence alignment and drafted the manuscript; AFG: participated in the immunoassays, and data analysis; PL: participated in the sequence alignment; YV: participated in animal and food strains isolation, BG: participated in human strains isolation JLS: conceived of the study, and participated in its design and coordination; JLK: designed the study, performed the statistical analysis, participated in its coordination, and helped to draft the manuscript; KSL: carried out the molecular genetic, performed data analysis, carried out the immunoassays and drafted the manuscript

All authors read and approved the final manuscript.
